# Parity and breastfeeding are protective against breast cancer in Nigerian women

**DOI:** 10.1038/sj.bjc.6604275

**Published:** 2008-02-26

**Authors:** D Huo, C A Adebamowo, T O Ogundiran, E E Akang, O Campbell, A Adenipekun, S Cummings, J Fackenthal, F Ademuyiwa, H Ahsan, O I Olopade

**Affiliations:** 1 Department of Health Studies, University of Chicago, Chicago, IL, USA; 2 Department of Surgery, University of Ibadan and University College Hospital, Ibadan, Nigeria; 3 Department of Pathology, University of Ibadan and University College Hospital, Ibadan, Nigeria; 4 Department of Radiotherapy, University of Ibadan and University College Hospital, Ibadan, Nigeria; 5 Department of Medicine, University of Chicago, Chicago, IL, USA

**Keywords:** African, breast cancer, case–control study, reproductive history, lactation

## Abstract

As the relation between reproductive factors and breast cancer risk has not been systematically studied in indigenous women of sub-Saharan Africa, we examined this in a case–control study in Nigeria. In-person interviews were conducted using structured questionnaires to collect detailed reproductive history in 819 breast cancer cases and 569 community controls between 1998 and 2006. Logistic regression was used to estimate odds ratios (ORs) and 95% confidence intervals (CI). Compared with women with menarcheal age <17 years, the adjusted OR for women with menarcheal age ⩾17 years was 0.72 (95% CI: 0.54–0.95, *P*=0.02). Parity was negatively associated with risk (*P-*trend=0.02) but age at first live birth was not significant (*P*=0.16). Importantly, breast cancer risk decreased by 7% for every 12 months of breastfeeding (*P*-trend=0.005). It is worth noting that the distribution of reproductive risk factors changed significantly from early to late birth cohorts in the direction of increasing breast cancer incidence. Our findings also highlight the heterogeneity of breast cancer aetiology across populations, and indicate the need for further studies among indigenous sub-Saharan women.

Nigeria is the most populous nation in Africa with about 140 million people, and one in five Black persons all over the world has Nigerian ancestry. Historically, Nigeria has a lower incidence rate of breast cancer compared with the United States and other developed countries (Parkin *et al*, 2005); however, the disease is the most common malignancy seen in Nigerian women and its incidence rate seems to be rising. Few studies have examined risk factors for breast cancer in Nigerian women and few have systematically examined the association with reproductive factors in sub-Saharan Africa (Adebamowo *et al*, 2003a, 2003b; Okobia *et al*, 2006). Early age at menarche was identified as a risk factor in one study (Adebamowo *et al*, 2003b) and late age at first full-term pregnancy was found to be significant in another study (Okobia *et al*, 2006). Because modest sample sizes in these studies might have limited the finding of associations with other reproductive factors, we have examined a wider range of such factors on breast cancer risk among Nigerian women in the expanded case–control study conducted in Ibadan, Nigerian from 1998 to 2006.

## MATERIALS AND METHODS

### Study sample

The study setting and design are described in detail elsewhere (Huo *et al*, 2007); it is an expansion of the case–control study of breast cancer in Ibadan, Nigeria (Adebamowo *et al*, 2003a, 2003b). Briefly, cases were identified through the Surgical Oncology and Radiotherapy units of the University College Hospital (UCH), Ibadan, which serves a population of 3 million and is a referral centre for other hospitals in the region. On the basis of referral patterns, the majority of breast cancer cases in the region are probably seen at UCH. All consecutive female cases aged 18 and above, with a histologic or clinical diagnosis of invasive breast cancer between March 1998 and July 2006, were eligible. Most eligible patients agreed to participate, with a refusal rate of only 4%.

During the period of case enrolment, a community adjoining the hospital was randomly selected by ballot from the list of all the communities in its hypothetical catchment area and considered to be stable, socio-economically diverse, and represents the diversity of UCH patients. Names were then randomly selected from the community register and the individuals were invited to visit a clinic setup in the community for the study. Inclusion criteria for the controls were female, aged 18 years or older, absence of any type of cancer, and ability to give informed consent.

### Data collection and measurements

The study was approved by the Institutional Review Boards of the University of Chicago and the University of Ibadan. After obtaining informed consent, trained nurse interviewers administered a structured questionnaire, measured height and weight, and obtained blood samples. The questionnaire covered demographic characteristics, family history of breast cancer and history of benign breast disease, lifestyle factors, menstrual and reproductive history, and hormonal contraceptive use.

We examined four reproductive factors in relation to breast cancer risk which are as follows: age at menarche, parity, age at first live birth, and duration of breastfeeding. Ages at menarche and parity were each considered in four categories: 10–14, 15–16, 17–18, and >18 years old and 0, 1–3, 4–6, and ⩾7 live births, respectively. Age at first live birth was grouped into four: <20, 20–24, 25–29, and 30 or older; lifetime duration of breastfeeding into five categories: ⩽24, 25–48, 49–72, 73–96, and >96 months. Since lifetime duration of breastfeeding tends to be longer as parity increases, mean duration of breastfeeding per child was calculated as total duration divided by parity for parous women, and it was dichotomised as < 12 and ⩾12 months.

On the basis of current literature, we considered the following as potential confounders and categorised as appropriate for further analysis using logistic regressions: age at diagnosis or interview (5-year interval categories), ethnicity (Yoruba, others), education (none, elementary, secondary, vocational, and some college or above), family history of breast cancer (yes, no), benign breast disease (yes, no), hormonal contraceptive use (ever, never), alcohol drinking (yes, no), height, body mass index (BMI), and menopausal status (premenopausal, natural postmenopausal, artificial postmenopausal). Alcohol drinking was defined as consumption of alcoholic beverages at least once a week for 6 months or longer.

### Statistical analyses

Demographic factors and potential confounding variables were compared between cases and controls using *t*-tests or Wilcoxon rank-sum tests for continuous variables and *χ*^2^ tests for categorical data. Logistic regression models were used to examine the relationship of breast cancer risk with reproductive factors. Odds ratio (OR) and 95% confidence intervals (CI) were computed as measures of association from the logistic models. Multiple logistic regression models were used to adjust for age and other potential confounders listed above. We also tested whether the effects of reproductive factors varied according to menopausal status by adding an interaction term in the logistic models. All five reproductive factors were included as both continuous and categorical variables in the models. Parity was further modelled using a linear spline function with a knot at one live birth to assess the effect of first live birth and trend after the first live birth, simultaneously (Greenland, 1998). Finally, linear regression or Wilcoxon rank-sum test was used to examine the secular trend of reproductive factors according to birth cohort of controls. Kaplan–Meier survival analysis was conducted to estimate the proportion having first baby at a specified age. All *P*-values reported are two-sided.

About 8% of participants had a missing value for age at menarche. Data were occasionally missing for other variables as well. To use all available information and avoid bias due to listwise deletion in the multivariate analysis, missing values were imputed 20 times via the method of multiple imputation by chained equations (van Buuren *et al*, 1999). Standard errors of regression coefficients were determined using a general formula for combining estimates in multiple imputation, which assumes that data are missing at random (Rubin, 1987). Older women tended to forget their menarcheal age. After including age in the multiple imputation models, it is reasonable to believe that the probability of missing menarcheal age is unrelated to the value itself. All statistical analyses were conducted with Stata 9.2 (StataCorp, College Station, TX, USA). Multiple imputation was conducted using the ice module (Royston, 2004).

## RESULTS

There were 1388 women in this study: 819 cases and 569 controls. Table 1 shows the demographic characteristics and nonreproductive factors in cases and controls. On average, cases were 5 years older than controls. Most participants were Yoruba (more so among the controls), the dominant group in Southwestern Nigeria, but there were also some Hausa and Ibo. Cases and controls were similar in hormonal contraceptive use, years of education, and BMI. Compared with controls, cases were more likely to have a family history of breast cancer, a history of benign breast disease, and to have consumed alcohol. More cases were postmenopausal women than controls were. Finally, cases were 1.7 cm, on average, taller than controls. All these variables were considered to be potential confounders and were adjusted in subsequent analyses.

Table 2 presents the distribution of reproductive factors for cases and controls. The majority of women had their menarche at or after age 15, and it was inversely associated with breast cancer risk (*P*=0.001). In the age-adjusted analysis, women with menarche at or after age 17 years showed about 40% lower risk than those with menarche at less than 17 years. Cases were more likely than controls to be nulliparous, and there was an inverse trend between parity and risk (*P*=0.02). In the spline regression, the age-adjusted OR was 0.69 (95% CI: 0.43–1.09) for the first live birth and 0.97 (95% CI: 0.91–1.03) for each later additional birth. No significant difference was found between cases and controls with regard to age at first live birth. All but four parous women (0.3%) had breastfed their babies. The lifetime duration of breastfeeding was quite long, averaging 66 months (range: 1–294 months) with overall, only 1.4% for less than 6 months. There was a dose–response relationship between lifetime duration and risk (*P*=0.005). Mean duration of breastfeeding per child was also negatively associated with risk (*P*=0.007). Women who had breastfed at least 12 months per child had their risk decreased by one-third.

Overall, there were 220 (1.1%) missing values of 14 variables in Tables 1 and 2 (excluding derivative variables) distributed among 151 women (10.9%, 119 cases, and 32 controls). One hundred and five (7.6%) had missing age at menarche, particularly among older women: the mean age was 43.9 and 52.6 years, respectively, for women who remembered and those who forgot menarcheal age (*P*<0.001). Women with missing data were included in the multivariate analysis to avoid unnecessary loss of power and potential bias. Twenty data sets were generated by multiple imputation such that the efficiency of estimating OR was greater than 99.5% for the fraction of missing information up to 0.08.

Table 3 shows multivariate-adjusted ORs for reproductive factors using data after multiple imputation. After adjustment for parity, age at first live birth, breastfeeding, and other potential confounders, the association between age at menarche and risk was attenuated but still statistically significant. The adjusted OR was 0.72 (95% CI: 0.54–0.95, *P*=0.02) for menarche at or after age 17 years, compared with age less than 17 years. In the multivariate analysis, there was still an inversed association between parity and risk (*P*=0.02). In the spline regression, the multivariate-adjusted OR was 0.81 (95% CI: 0.49–1.34) for the first live birth and 0.95 (95% CI: 0.88–1.02) for each additional birth. Since there was a strong correlation between lifetime duration of breastfeeding and parity (*r*=0.83, *P*<0.001), the association between multiparity (in parous women) and risk was further examined after adjusting for duration of breastfeeding: the small beneficial effect disappeared with OR=1.04 (95% CI: 0.93–1.15, *P*=0.49) for each additional birth beyond the first. Conversely, the association between breast cancer and lifetime duration of breastfeeding persisted after adjustment for parity, age at first live birth, and other potential confounders (7% decline in OR for each 12 months breastfeeding; *P*=0.005). Similarly, mean duration of breastfeeding per child was negatively associated with risk: women who had breastfed at least 12 months per child had one-quarter decreased risk (*P*=0.06). Age at first live birth remained nonsignificant in the multivariate analyses. We also tested whether the effect of these reproductive factors varied by menopausal status and found no significant interaction between these factors and menopausal status (data not shown).

To examine how the prevalence of reproductive factors changed for women born in earlier and later decades, we grouped healthy controls into five birth cohorts as shown in Table 4. The average age at menarche decreased over time from 54% before age 17 years for women born before 1940, compared with 77% for those born after 1970. The numbers of pregnancies and live births were also significantly decreased over time among all women or those 40 years or older at interview. Because almost all babies (95%) were born to women younger than age 40, the number of live births in women 40 years or older represent their lifetime parity and younger women might not have reached their full potential for reproduction. The mean age at first live birth did not change substantially over time for parous women but there are more nulliparous women in the recent birth cohort. Taking into account nulliparous women who have not had their first baby by age 25, the proportion having their first baby before age 25 was significantly decreased from 73% in the oldest cohort to 36% in the youngest cohort. The lifetime duration of breastfeeding decreased markedly over time, partly due to smaller family size and partly due to shorter duration of breastfeeding per child. On the basis of ORs in Table 3 and the secular trend of the reproductive factors in Table 4 (excluding the youngest birth cohort), we estimated the contribution to the change in breast cancer incidence. Breast cancer incidence was estimated to have increased 7% due to change in menarcheal age, 15% due to decreased parity, and 15% due to shortened duration of breastfeeding per child.

## DISCUSSION

This study demonstrates that reproductive factors play an important role in breast cancer aetiology among indigenous African women. We found that ages at menarche, parity, and breastfeeding were significant predictors, although their distributions in our population were quite different from those in industrialised countries. We did not find that early age at first live birth was associated with decreased risk of breast cancer after extensive statistical adjustment.

Later age at menarche has been consistently associated with decreased risk of breast cancer in both premenopausal and postmenopausal women (Kelsey *et al*, 1993; Clavel-Chapelon and Gerber, 2002). In the current study, a delay of 2 years in menarche corresponds to a 7% reduction in risk, which is in line with the 10% reduction observed in an international multicentre study (Hsieh *et al*, 1990).

Parous women have a lower risk than nulliparous women, but the relationship between parity and breast cancer is complex (Kelsey *et al*, 1993; Colditz *et al*, 2006). Risk initially increases after the first pregnancy, then decreases after 10–15 years (Lambe *et al*, 1994). We observed the long-term protective effect, with 19% risk reduction for the first birth, but not the transient increase after the first birth, perhaps because on the modified Pike model, this was shorter for multiparous women (Rosner *et al*, 1994) about 80% of participants and were multiparous.

There is controversy over whether the number of full-term pregnancies beyond the first is protective (Kelsey *et al*, 1993). According to the collaborative reanalysis of 47 epidemiologic studies, each birth reduces the relative risk of breast cancer by 7% in the absence of breastfeeding and each child breastfed corresponds to a 3.4% decreased risk, but the association with parity was not significant after stratification by lifetime duration of breastfeeding (Collaborative Group on Hormonal Factors in Breast Cancer, 2002). Similarly, we found risk decreased by 5% per each additional birth after the first birth and by 7% per 12 months of breastfeeding. The beneficial effect of breastfeeding was independent of multiparity but multiparity was not an independent factor.

We did not find significant association between age at first live birth and risk, which seems inconsistent with all the evidence from studies mainly in Caucasian and Asian populations (Kelsey *et al*, 1993; Clavel-Chapelon and Gerber, 2002). Risk factors may vary by breast cancer subtypes, as it is a heterogeneous disease, varying in different populations. A recent meta-analysis showed that the age at first birth was associated only with ER+PR+ breast cancer but not with ER−PR− breast cancer (Ma *et al*, 2006). In addition, a large population-based study in the United States showed that ER+ or PR+ breast tumours are the majority, although proportions varied by race/ethnicity: the highest in non-Hispanic White or Asian Americans and Pacific Islanders, followed by Hispanics, and the lowest in African Americans (Chu and Anderson, 2002). In contrast, two studies in Nigeria (Ikpatt and Ndoma-Egba, 2003; Gukas *et al*, 2005) and a collaborative study in West Africa (OI Olopade *et al*, unpublished data) found that only about 25% of tumours were ER+, though a recent study in Nigeria found that 65% were ER+ (Adebamowo *et al*, 2007). Therefore, the lack of association between age at first live birth and breast cancer risk may be due to heterogeneity of tumour subtypes in our study sample.

The current study documented that 30% of Nigerian women started menarche after age 16, the majority had multiple live births, they almost always breastfed their babies, more than half for 12 months or longer per child. These factors probably contribute to the low incidence of breast cancer in Nigerian women. However, these protective effects may be diminishing as women adopt a more western lifestyle. In community controls, their patterns should show similar changes to those in the general population. We found that all reproductive factors changed in the direction towards increased breast cancer risk. We estimated that risk increased by about 37% in women born in 1960s compared to those born in 1930s, due to change in menarcheal age, parity, and duration of breastfeeding. Therefore, incidence in Nigerian population might have increased in the past three decades, and we speculate that it will continue as women born after 1960s become older. A systematic study of the secular trend of incidence in Nigeria is of public health importance.

Several limitations are relevant. First, reproductive history was self-reported and may be subject to recall bias. Women can reliably report the number of children they have had, so differential reporting of parity is probably not a serious problem. Duration of breastfeeding, age at menarche, and age at first live birth are less accurate, but presumably not differentially recalled by cases and controls. Second, although the multiple imputation method was employed reasonably to address the missing age at menarche, additional ‘measurement error’ is unavoidable. Third, the estimated trend of breast cancer incidence in Nigeria may have limited accuracy because it is based on the reproductive histories of women from a single community, relative risks from this single study, and the assumption that the population structure is constant over time.

Our study population provides an important opportunity to elucidate the effect of reproductive risk factors on risk in prevalence ranges that are not seen in the developed world. A live birth, extended breastfeeding, and later onset of menstruation are protective in Nigerian women, but we found no independent effect of age at first live birth. The study also documents the changing pattern in reproductive factors, which could explain the generally low incidence of breast cancer in Nigerian women and suggests that this may increase over time.

## Figures and Tables

**Table 1:** Selected characteristics of cases with invasive breast cancer and community controls, Nigeria, 1998–2006

**Characteristic**	**Cases (*n*=819)**	**Controls (*n*=569)**	***P*-value**
Age in years, mean±s.d.	46.8±11.3	41.2±13.8	<0.001
			
*Ethnicity, n* (%)			
Yoruba	609 (74.4)	537 (94.4)	<0.001
Ibo	89 (10.9)	12 (2.1)	
Hausa	14 (1.7)	1 (0.2)	
Others	107 (13.1)	19 (3.3)	
			
*Education, n* (%)			0.13
No formal	191 (23.3)	112 (19.7)	
Elementary	197 (24.1)	106 (18.6)	
Secondary	131 (16.0)	160 (28.1)	
Vocational	123 (15.0)	63 (11.1)	
Some college or above	177 (21.6)	128 (22.5)	
			
*Family history of breast cancer, n* (%)			
Yes	69 (8.4)	27 (4.7)	0.01
No	750 (91.6)	542 (95.3)	
			
*Benign breast disease, n* (%)			
Yes	141 (17.2)	71 (12.5)	0.02
No	678 (82.8)	498 (87.5)	
			
*Menopausal status, n* (%)			<0.001
Premenopausal	463 (56.5)	404 (71.0)	
Postmenopausal, natural	339 (41.4)	161 (28.3)	
Postmenopausal, artificial	17 (2.1)	4 (0.7)	
Age at natural menopause, mean±s.d.	48.2±5.7	49.9±5.5	0.002
			
*Hormonal contraceptives, n* (%)			
Yes	198 (24.2)	125 (22.0)	0.37
No	621 (75.8)	444 (78.0)	
			
*Alcohol drink, n* (%)			
Yes	107 (13.1)	43 (7.6)	0.001
No	708 (86.9)	526 (92.4)	
Height in cm, mean±s.d.	160.2±6.8	158.5±6.6	<0.001
BMI in kg m^−2^, mean±s.d.	25.4±5.4	25.2±5.5	0.65

Abbreviations: BMI=body mass index; s.d.=standard deviation.

**Table 2:** Reproductive factors and breast cancer, Nigeria, 1998–2006

	**No. of cases**	**No. of controls**	**Age-adjusted OR (95% CI)**
*Age at menarche (years)*			
10–14	265	181	1.0 (ref.)
15–16	300	203	0.90 (0.68–1.20)
17–18	123	120	0.60 (0.43–0.83)
⩾19	47	44	0.56 (0.35–0.91)
*P-*value for trend			*0.001*
Mean±s.d.	15.2±2.1	15.4±2.3	
Per 2-year delay			0.86 (0.77–0.96)
			
*Parity*			
0	74	103	1.0 (ref.)
1–3	247	175	0.70 (0.45–1.11)
4–6	361	207	0.55 (0.34–0.89)
⩾7	135	74	0.52 (0.30–0.89)
*P-*value for trend			*0.02*
Mean±s.d.	4.2±2.5	3.5±2.6	
First live birth			0.69 (0.43–1.09)
Each additional live birth			0.97 (0.91–1.03)
			
*Age at first live birth (years)* a			
<20	166	84	1.0 (ref.)
20–24	325	206	0.75 (0.54–1.04)
25–29	181	132	0.70 (0.49–1.00)
⩾30	66	32	0.94 (0.57–1.57)
*P-*value for trend			*0.40*
Mean±s.d.	22.9±4.7	23.2±4.1	
Per 5-year increase			0.93 (0.81–1.07)
			
*Lifetime duration of breastfeeding (months)* a			
⩽24	130	82	1.0 (ref.)
25–48	198	115	0.82 (0.56–1.21)
49–72	147	100	0.59 (0.39–0.90)
73–96	125	71	0.63 (0.40–0.99)
> 96	142	88	0.54 (0.34–0.85)
*P-*value for trend			*0.005*
Mean±s.d.	66±44	65±44	
Per 12-month increase			0.96 (0.92–0.99)
			
*Mean duration of breastfeeding per child (months)* a			
<12	234	113	1.0 (ref.)
⩾12	509	343	0.68 (0.52–0.90)
*P-value*			*0.007*

Abbreviations: CI=confidence interval; OR=odds ratio; s.d.=standard deviation.

a Among parous women.

**Table 3:** Multiple logistic regression analysis using data after multiple imputation on the relationship between reproductive factors and breast cancer, Nigeria, 1998–2006

	**OR (95% CI)^a^**	**OR (95% CI)**
*Age at menarche (years)*		
10–14	1.0 (ref.)	1.0 (ref.)^b^
15–16	0.99 (0.73–1.34)	1.00 (0.73–1.35)
17–18	0.71 (0.49–1.02)	0.71 (0.50–1.03)
⩾19	0.68 (0.41–1.14)	0.71 (0.42–1.20)
*P-*value for trend	*0.03*	*0.04*
Per 2-year delay	0.90 (0.80–1.02)	0.92 (0.81–1.03)^b^
		
*Parity*		
0	1.0 (ref.)	1.0 (ref.)^c^
1–3	0.78 (0.48–1.27)	0.79 (0.48–1.29)
4–6	0.63 (0.38–1.06)	0.63 (0.37–1.06)
⩾7	0.51 (0.28–0.93)	0.52 (0.28–0.94)
*P-*value for trend	*0.02*	*0.02*
First live birth	0.80 (0.49–1.32)	0.81 (0.49–1.34)^c^
Each additional live birth	0.95 (0.88–1.02)	0.95 (0.88–1.02)^c^
		
*Age at first live birth in parous women (years)*		
<20	1.0 (ref.)	1.0 (ref.)^d^
20–24	0.88 (0.62–1.26)	0.87 (0.60–1.25)
25–29	0.73 (0.48–1.09)	0.66 (0.43–1.02)
⩾30	0.90 (0.51–1.58)	0.80 (0.44–1.47)
*P-*value for trend	*0.32*	*0.16*
Per 5-year increase	0.92 (0.78–1.08)	0.87 (0.73–1.04)^d^
		
*Lifetime duration of breastfeeding in parous women (months)*		
⩽24	1.0 (ref.)	1.0 (ref.)^e^
25–48	0.94 (0.62–1.43)	0.83 (0.54–1.30)
49–72	0.66 (0.42–1.05)	0.52 (0.31–0.89)
73–96	0.73 (0.44–1.20)	0.53 (0.28–0.98)
>96	0.57 (0.34–0.95)	0.36 (0.17–0.75)
*P-*value for trend	*0.02*	*0.005*
Per 12-month increase	0.96 (0.92–1.00)	0.93 (0.87–1.00)^e^
		
*Mean duration of breastfeeding per child in parous women (months)*		
<12	1.0 (ref.)	1.0 (ref.)^f^
⩾12	0.75 (0.56–1.02)	0.74 (0.55–1.01)
*P-value*	*0.07*	*0.06*

Abbreviations: CI=confidence interval; OR=odds ratio; s.d.=standard deviation.

a Adjusted for age, ethnicity, education, family history of breast cancer, benign breast disease, hormonal contraceptive use, alcohol drinking, height, body mass index, and menopausal status.

b Additionally adjusted for parity, age at first live birth, and duration of breastfeeding.

c Additionally adjusted for age at menarche.

d Additionally adjusted for age at menarche, parity, and duration of breastfeeding.

e Additionally adjusted for age at menarche, parity, and age at first live birth.

f Additionally adjusted for age at menarche and age at first live birth.

**Table 4:** Reproductive factors by birth cohort in healthy controls, Nigeria, 1998–2006

	**<1940 (*n*=46)**	**1940–1949 (*n*=75)**	**1950–1959 (*n*=117)**	**1960–1969 (*n*=145)**	**⩾1970 (*n*=186)**	***P*-value**
*Age at menarche (years), n* (%)						
<17	22 (53.7)	42 (59.2)	73 (66.4)	106 (74.6)	141 (76.6)	<0.001
⩾17	19 (46.3)	29 (40.8)	37 (33.6)	36 (25.4)	43 (23.4)	
Mean±s.d.	16.4±2.6	16.2±2.5	15.6±2.4	15.1±2.2	15.0±2.1	<0.001
						
*No. of pregnancy, mean±s.d.*						
In all women	7.0±2.8	6.5±1.9	5.6±1.9	4.2±2.0	1.4±1.6	<0.001
In women ⩾40 years old	7.0±2.8	6.5±1.9	5.6±1.9	4.6±2.1	—	<0.001
						
*Parity, mean±s.d.*						
In all women	6.1±2.5	5.8±1.8	5.0±1.9	3.5±1.7	1.0±1.2	<0.001
In women ⩾40 years old	6.1±2.5	5.8±1.8	5.0±1.9	4.0±1.7	—	<0.001
						
*Age at first live birth (years)*						
<25, %	72.7	63.0	66.7	56.7	35.6^a^	<0.001
Mean±s.d.	22.3±4.0	23.4±3.7	22.7±4.1	23.8±4.4	23.4±3.9	0.11
						
*Lifetime duration of breastfeeding (months), mean±s.d.*						
In all women	117±61	88±43	71±35	56±30	27±17	<0.001
In women ⩾40 years age	117±61	88±43	71±35	64±34	—	<0.001
Mean duration of breastfeeding per child (months), mean±s.d.	18.8±7.5	15.1±6.7	13.8±4.5	14.5±4.1	13.6±4.7	<0.001

Abbreviation: s.d.=standard deviation.

a Calculated from Kaplan–Meier analysis, which took into account women younger than 25 years.
